# Thinking About the Future: A Review of Prognostic Scales Used in Acute Stroke

**DOI:** 10.3389/fneur.2019.00274

**Published:** 2019-03-21

**Authors:** Bogna A. Drozdowska, Sarjit Singh, Terence J. Quinn

**Affiliations:** Institute of Cardiovascular and Medical Sciences, College of Medical, Veterinary & Life Sciences, University of Glasgow, Glasgow, United Kingdom

**Keywords:** stroke, functional outcome, prognostic scale, risk score, validation

## Abstract

**Background:** There are many prognostic scales that aim to predict functional outcome following acute stroke. Despite considerable research interest, these scales have had limited impact in routine clinical practice. This may be due to perceived problems with internal validity (quality of research), as well as external validity (generalizability of results). We set out to collate information on exemplar stroke prognosis scales, giving particular attention to the scale content, derivation, and validation.

**Methods:** We performed a focused literature search, designed to return high profile scales that use baseline clinical data to predict mortality or disability. We described prognostic utility and collated information on the content, development and validation of the tools. We critically appraised chosen scales based on the CHecklist for critical Appraisal and data extraction for systematic Reviews of prediction Modeling Studies (CHARMS).

**Results:** We chose 10 primary scales that met our inclusion criteria, six of which had revised/modified versions. Most primary scales used 5 input variables (range: 4–13), with substantial overlap in the variables included. All scales included age, eight included a measure of stroke severity, while five scales incorporated pre-stroke level of function (often using modified Rankin Scale), comorbidities and classification of stroke type. Through our critical appraisal, we found issues relating to excluding patients with missing data from derivation studies, and basing the selection of model variable on significance in univariable analysis (in both cases noted for six studies). We identified separate external validation studies for all primary scales but one, with a total of 60 validation studies.

**Conclusions:** Most acute stroke prognosis scales use similar variables to predict long-term outcomes and most have reasonable prognostic accuracy. While not all published scales followed best practice in development, most have been subsequently validated. Lack of clinical uptake may relate more to practical application of scales rather than validity. Impact studies are now necessary to investigate clinical usefulness of existing scales.

## Introduction

Outcomes following a stroke event can range from full recovery, through varying degrees of disability to death. Given the subsequent need for intervention planning, resource use, and lifestyle adjustments, predicting outcome following stroke is of key interest and importance to patients, their families, clinicians, and hospital administrators. Various tools exist to assist in estimating stroke-related prognosis. For example, the ABCD2 score uses clinical features to predict risk of stroke following transient ischemic attack (TIA) (1). Although there are criticisms of ABCD2, it is widely used and included in stroke guidelines (2).

Scales for predicting acute stroke outcomes from baseline features are also described in the scientific literature (3–5). Often prognosis scales report mortality; however, given the disabling nature of stroke, scales predicting death and/or longer-term disability may be more useful in the stroke setting (6). However, these prognostic scales have had limited clinical traction and have not been incorporated into routine clinical practice (3). There are many plausible reasons why these scales have not been adopted by the stroke community (6). In an acute setting, scales may be perceived as being too complex to use or may require information that is not routinely available (for example, sophisticated neuroimaging) (3). Clinicians may moreover be concerned that scales are inherently too generic, and may not provide insight over what the clinician can conclude based on individual patient factors and clinical gestalt (7).

For many scales, clinicians may simply not be convinced of their utility or the rigor of the underpinning science. These points can be addressed by describing the validity of the scales. Issues with validity could relate to the methodological quality of the initial derivation of the scale (internal validity) or the generalizability of a scale to a real-world population (external validity). Robust evidence of validity requires assessment of the scale in cohorts independent of the population used to derive that scale (8). However, In some areas of stroke practice, for example rehabilitation, it has been demonstrated that independent validation studies are lacking for many scales (5).

Collating evidence around the quality of the research that led to development of prognostic scales and also the results of subsequent validation work could be useful for various stakeholders. For clinicians it may convince of the utility, or lack of utility, of certain tools; for researchers it may point to common methodological limitations that need to be addressed in future work and for policy developers, if a certain tool has a more compelling evidence base than others, then this scale may be preferred in guidelines.

Previous reviews have reported that many stroke prognosis scales have similar properties such as discrimination and calibration. These reviews also highlight the limited evidence for external validity of many commonly used stroke scales (9, 10). Distinguishing an optimal prognostic tool may not be possible based on psychometric properties alone and factors such as feasibility and acceptability in the real world setting need to be considered.

We sought to collate and appraise a selection of exemplar published stroke scales, designed for use in acute care settings. We used these as a platform to discuss methodological quality of prognostic scale development, while also considering potential barriers or facilitators to implementation of the scales in clinical practice.

## Methods

We performed a focused review of the literature to find scales predicting post-stroke mortality and/or function. Our approach followed that used in a recent comparative efficacy review of stroke scales (9). Rather than assess every tool that has ever been used to make outcome predictions in stroke, we were interested in examples of high profile prognostic scales. Although our intention was not a comprehensive search, we followed, where relevant, Preferred Reporting Items for Systematic Reviews and Meta-Analyses (PRISMA) guidance for designing and reporting our study (11). For consistency in use of terminology, we have referred to the prediction models as “scales,” and the calculated outputs of models as “scores.”

### Inclusion/Exclusion Criteria

We defined a scale as any tool that uses more than two determinants to estimate the probability of a certain outcome. We focused on scales with predominant clinical input variables that can be applied without specialist resources or tests and to this end we excluded scales that had more than two neuroimaging input variables. We limited to ischemic or all cause (undifferentiated) stroke scales, recognizing the differing natural progression of ischemic and hemorrhagic strokes.

### Search Strategy

Our focus was on scales that are well known in the stroke field and so we adapted our search using an approach that has been used in other focused stroke studies (12, 13). We limited our search to 11 high profile, international journals, chosen based on relevance to stroke and clinical impact, covering fields of stroke, neurology, internal medicine, and geriatric medicine (a full list of journals and the search strategy are included in Supplementary Materials).

Searches were from inception to May 2018. Once we had selected chosen scales we used PUBMED and Google Scholar electronic search engines to find the initial development paper and any potential validation papers. A single researcher (SS) performed the search and screened the results We assessed internal validity of the search results by screening title lists twice (October 2015 and May 2018).

### Data Extraction and Critical Appraisal

Two researchers (BD, SS) extracted data from selected studies, using a pre-specified proforma. This included information on: data source, study sample characteristics, predictor, and outcome variables, procedures involved in model derivation, methods of validation, measures of performance and presentation of results. Extracted data were comprehensively reviewed to inform critical appraisal, a process in which all authors (BD, SS, TQ) were involved.

The methodological assessment of prognostic scales is an evolving landscape. Although there is no consensus preferred approach to this, there are certain features common to most tools that purport to assess validity of prognosis research. We based our assessment on recommendations from the Critical Appraisal and Data Extraction for Systematic Reviews of Prediction Modeling Studies (CHARMS) checklist (14). Discrepancies in assessment between researchers were discussed and resolved through consensus.

#### Data Used for Scale Development

We assessed the representativeness of the sample from which information was collected. Generalizability of a scale to a broader patient population may be compromised when recruitment takes place in a highly specific context or is limited to a relatively homogeneous group; when multiple inclusion and exclusion criteria are applied; and finally, when patients with missing data are excluded from the study (complete-case analysis). The latter presents itself as an issue, as it is uncommon for variable values to be missing completely at random. Often this is related to other predictors, the outcome, or even the value of that variable itself (15). Therefore, patients with missing data are likely to form a selective rather than random subsample of the initial baseline cohort, and may substantially differ from those included in the analysis (14, 16).

#### Scale Variables

For predictor and outcome variables, a particular concern was whether they are precisely defined and measured in a way that can be reproduced across different centers. It is recommended that continuous data (e.g., age) are not categorized when introduced to a model as a predictor (17, 18). Doing so is associated with loss of information and power, and increases the risk of generating inaccurate estimates and residual confounding. Finally, bias may arise from lack of blinding to predictors when assessing an outcome, or blinding to the outcome where predictors are assessed retrospectively.

#### Scale Development Process

In this context, we assessed study sample size against the number of candidate predictors being tested. For logistic regression procedures, we considered a minimum of ten events (number of patients with the less frequent outcome) per variable to be sufficient (19, 20). Evaluating the selection process of predictors for inclusion in scales presents a challenge, as there is no agreed approach (21). There are however certain practices that are consistently stated to increase risk of bias. One is selecting predictors for inclusion in multivariable analysis based on significance in univariable analyses (22). This approach may lead to exclusion of predictors that could be associated with the outcome after adjusting for the effects of other factors. A data-driven approach to variable selection may lead to model overfitting (23) and forward selection techniques should be avoided in multivariable modeling (24). Either a full model approach (all candidate variables included in the model) or backwards elimination (beginning with all candidate predictors, removing those that do not satisfy a pre-specified statistical criterion) is preferable (25).

#### Assessment of Scale Performance

We distinguished three levels of validation: apparent, internal and external (26–28). In apparent validation, predictive ability is assessed in the development set itself and may give overoptimistic performance estimates. With internal validation two approaches are described, split-sample and cross-validation. These involve randomly splitting the baseline sample into development and assessment sets. In the split-sample technique, the population is divided once, in cross-validation, the process of sample division is repeated for consecutive fractions of subjects, thus allowing for each participant to be included in the validation set once. Here, a larger part of the baseline sample can be used for model derivation, avoiding the considerable loss of power associated with split sample approaches (14). The most efficient method of internal validation is considered to be bootstrapping, where samples are drawn with replacement from the original dataset, replicating sampling from an underlying population (26). The generated sample is of the same size as the original dataset.

Importantly, even with use of internal validation techniques, assessing a scale's performance in the development cohort is considered insufficient to confirm its value and general applicability (27, 29), In view of this, we prioritized findings from external validation studies. External data can differ from derivation data in terms of when and where it was collected, as well as by research group. Typically, an external dataset is comparable to the original, however in some studies a model is intentionally tested in a population characterized by different clinical features.

Reviewing study results on predictive performance, we focused on measures of discrimination and calibration, as these properties are necessary (although not sufficient) to ensure clinical usefulness of a prognostic scale (14, 27). Discrimination relates to the ability of a model to accurately distinguish between those who develop a certain outcome and those who do not, and is commonly expressed as the area under the receiver operating characteristic curve (AUROC) (30). To aid interpretation of results, we applied to following AUROC cut-off values: 1.00—perfect discrimination; 0.90 to 0.99—excellent; 0.80 to 0.89—good; 0.70 to 0.79—fair; 0.51 to 0.69—poor; 0.50—of no value, equivalent to chance (31). Calibration refers to the level of agreement between observed and predicted outcome probabilities, with assessment preferably based on inspection of calibration plots/curves (32). Graphical evaluation can be accompanied by reporting results of the Hosmer-Lemeshow test, assessing whether there is a significant difference between observed and predicted outcomes. The test has however limited power for detection of poor calibration, is oversensitive in large samples, and does not allow to determine the direction of miscalibration (33).

## Results

### Overview of Scale Content and Quality

Our search returned 3817 results. We found 10 primary scales that met our inclusion criteria, six of which also had modified versions published (Tables 1, 2). Scales used from four to thirteen input variables, with a mode of five. There was considerable overlap in the predictors used, including variables relating to demographics, past medical history and the acute stroke event (Table 3). The most commonly incorporated predictors were: age (all ten scales), a measure of stroke severity (eight); pre-stroke function, comorbidities and stroke subtype (each present in five scales). Individual scale content, with scoring, is presented in Table 4.

**Table 1:** Derivation study characteristics.

**Scale**	**Derivation dataset**	**Location**	**Recruitment type**	**Enrolment dates**	**Stroke type**	**Exclusion criteria of note**
ASTRAL	Acute Stroke Registry and Analysis of Lausanne	Switzerland	Single-center	Jan 2003 to Jul 2010	Ischemic	Pre-stroke mRS > 2
DRAGON	Bespoke cohort	Finland	Single-center	1995 to Sep 2010	Ischemic	Basilar artery occlusions
FSV	Stroke Outcome Study	Canada	Single-center	2001 to 2002	Ischemic, hemorrhagic	
iSCORE	Registry of the Canadian Stroke Network	Canada	Multi-center	Jul 2003 to Jun 2008	Ischemic	
PLAN	Registry of the Canadian Stroke Network	Canada	Multi-center	Jul 2003 to Mar 2008	Ischemic	Patients receiving IV-tPA
SNARL	Endovascular registry	Unclear	Multi-center	Sep 2009 to Jul 2011	Ischemic	
SOAR	Anglia Stroke and Heart Clinical Network Database	United Kingdom	Multi-center	1997 to 2010	Ischemic, hemorrhagic	
SPI	Carotid ultrasound register	United States	Single-center	Jan 1984 to Feb 1987	Ischemic	Artificial heart valves, previous cerebrovascular event
S-TPI	NINDS 1 & 2, ATLANTIS A & B, ECASS 2 cohorts	International	Multi-center	Jan 1991 to Jul 1998^*^	Ischemic	Multiple, related to primary randomized control trials; minor strokes
THRIVE	MERCI, Multi MERCI cohorts	International	Multi-center	May 2001 to Dec 2003; Jan 2004 to Jul 2006	Ischemic	Multiple, related to primary single-arm trials.

*IV-tPA, intravenous tissue plasminogen activator; mRS, modified Rankin Scale*.

* *Combined recruitment period for all trials*.

**Table 2:** Derivation study participants, outcomes and scale discriminatory power.

**Scale**	**Baseline sample size**	**Total sample used for model derivation (% of baseline sample)**	**Outcome**	**Timepoint of outcome assessment**	**Number of patients with outcome (%)**	AUROC (95% CI)^*^ for apparent/internal validation
ASTRAL	1,967	1,645 (84%)	mRS 3–6	3 months	559 (34%)	0.85
DRAGON	1,529	1,319 (86%)	mRS 0–2 mRS 3–4 mRS 5–6	3 months 3 months 3 months	798 (60%) 339 (26%) 182 (14%)	Not reported Not reported 0.84 (0.80 – 0.87)
FSV	598	538 (90%)	mRS 0–1 mRS 0–2 mRS 5–6	6 months 6 months 6 months	Not reported	0.86 0.89 0.87
iSCORE	Not available	8,223 (n/a)	Death Death	1 month 1 year	1,004 (12.2%) 1,853 (22.5%)	0.85^**^ 0.82^**^
PLAN	12,576	4,943 (39%)	Death Death mRS 5–6	1 month 1 year Discharge	569 (11.5%) 1,088 (22.0%) 735 (14.9%)	0.85 (0.84–0.87) 0.82 (0.81–0.84) 0.89 (0.87–0.90)
SNARL	556	511 (92%)	mRS 0–2	3 months	186 (36.4%)	0.79 (0.75–0.83)
SOAR	Not available	12,355 (n/a)	Death Death LOS < 8 days	7 days Inpatient n/a	Not reported Not reported n/a	0.79 (0.78–0.80) 0.79 (0.78–0.80) 0.61 (0.60–0.62)
SPI	352	142 (40%)	Stroke or death	2 years	38 (27%)	Not reported
S-TPI	2,184	2,131 (98%)	mRS 0–1 mRS 5–6	3 months 3 months	773 (36%) 464 (22%)	0.79^**^ 0.78^**^
THRIVE	305	Not reported	mRS 0–2 Death	3 months 3 months	94 (n/a) 111 (n/a)	0.71^**^ Not reported

*mRS, modified Rankin Scale; LOS, length of hospital stay*.

* *Where reported in derivation study*.

** *AUROC for model, not derived risk score*.

**Table 3:** Categories of input variables included in post-stroke prognostic scales.

**Scale**	**Age**	**Sex**	**Pre-stroke functional status**	**Comorbidity**	**Time from symptom onset**	**Acute physiology**	**Stroke severity**	**Stroke classification**	**Imaging findings**	**Treatment**
Astral										
Dragon										
FSV										
iScore										
PLAN										
SNARL										
SOAR										
SPI										
S-TPI										
THRIVE										

*Green fill denotes inclusion of a variable from the given category in a prognostic scale*.

**Table 4:** Scale variables and scoring systems.

**Scale**	**Variable**	**Level/category**	**Score**
ASTRAL	Acute glucose	< 3.7 or >7.3 mmol/L	1
	Age	Per every 5 years	1
	Any stroke-related visual field defect	Yes	2
	Level of consciousness	Decreased	3
	Symptom onset to treatment time	>3 hours	2
	Stroke severity (NIHSS)	Per every point	1
DRAGON	Hyperdense cerebral artery or early infarct signs on CT scan	None Either	0 1
		Both	2
	Pre-stroke functioning (mRS)	≤ 1 >1	0 1
	Age	< 65 years	0
		65–79 years	1
		>79 years	2
	Acute glucose	≤ 8 mmol/L >8 mmol/L	0 1
	Symptom onset to treatment time	≤ 90 min >90 min	0 1
	Stroke severity (NIHSS)	0–4	0
		5–9	1
		10–15	2
		>15	3
FSV	Able to lift both arms	Yes	2
	Able to walk unaided	Yes	1
	Age	< 80 years	1
	Verbal GCS	Normal	1
	Pre-stroke functioning (mRS)	Per every level	−1
FSV_DEV_	Able to lift both arms	No	2
	Age	≥80 years	3
	Verbal GCS	Abnormal	3
	Pre-stroke functioning (mRS)	Per every level	1
	Stroke subtype classification (OCSP)	TACS	2
iSCORE; 1-month outcome	Age	Per every year	1
	Sex	Male	10
	Stroke severity (CNS)	0	105
		≤ 4	65
		5-7	40
		>8	0
	Stroke subtype (TOAST)	Lacunar	0
		Non-lacunar	30
		Undetermined	35
	History of atrial fibrillation	Yes	10
	History of congestive heart failure	Yes	10
	Cancer	Yes	10
	Renal dialysis	Yes	35
	Pre-stroke functioning	Dependent	15
	Acute glucose	≥7.5 mmol/L	15
iSCORE; 1-year outcome	Age	Per every year	1
	Sex	Male	5
	Stroke severity (CNS)	0	70
		≤ 4	40
		5-7	25
		>8	0
	Stroke subtype (TOAST)	Lacunar	0
		Non-lacunar	15
		Undetermined	20
	History of atrial fibrillation	Yes	5
	History of congestive heart failure	Yes	10
	Previous myocardial infarction	Yes	5
	Current smoker	Yes	5
	Cancer	Yes	15
	Renal dialysis	Yes	40
	Pre-stroke functioning	Dependent	20
	Acute glucose	≥7.5 mmol/L	10
PLAN	Pre-stroke functioning	Dependent	1.5
	Cancer	Yes	1.5
	History of congestive heart failure	Yes	1.0
	History of atrial fibrillation	Yes	1.0
	Level of consciousness	Reduced	5.0
	Age	Per decade	1.0
	Arm weakness	Significant/total	2.0
	Leg weakness	Significant/total	2.0
	Neglect or aphasia	Either/both	1.0
SNARL	Symptomatic hemorrhage	No	2
	Stroke severity (NIHSS)	>20	0
		10–20	1
		< 10	3
	Age	>80 years	0
		60–79 years	1
		< 60 years	2
	Reperfusion (TICI)	≥2b	3
	Location of occlusion	M2 or distal	1
SOAR	Age	≤ 65	0
		66–85	1
SNARL		>85	2
	Stroke type	Hemorrhagic	1
	Stroke subtype classification (OCSP)	LACS	0
		PACS	0
		POCS	1
		TACS	2
	Pre-stroke functioning (mRS)	≤ 2	0
		3–4	1
		5	2
SPI	Age	>65	3
	Diabetes mellitus	Yes	3
	Acute severe hypertension	Yes	2
	Type of cerebrovascular event	TIA	0
		Stroke	2
	History of coronary heart disease	Yes	1
S-TPI; Good outcome	IV-tPA treatment	Yes	N/a^*^
	Age	Per every year	N/a^*^
	Acute systolic blood pressure	Per every 1 mmHg	N/a^*^
	Diabetes	Yes	N/a^*^
	Sex	Male	N/a^*^
	Stroke severity (NIHSS)	Per every point	N/a^*^
	Previous stroke	Yes	N/a^*^
	Symptom onset to treatment time	Per every minute	N/a^*^
	Treatment × systolic blood pressure		N/a^*^
	Treatment × sex		N/a^*^
	Treatment × previous stroke		N/a^*^
	Treatment × symptom onset to treatment time		N/a^*^
	Age × stroke severity		N/a^*^
S-TPI; Poor outcome	Age	Per every year	N/a^*^
	Stroke severity (NIHSS)	Per every point	N/a^*^
	Acute glucose	Per every mmol/L	N/a^*^
	ASPECTS score^**^	Per every point	N/a^*^
THRIVE	Age	≤ 59	0
		60–79	1
		≥80	2
	Stroke severity (NIHSS)	≤ 10	0
		11–20	2
		≥80	4
	Diabetes mellitus	Yes	1
	History of hypertension	Yes	1
	History of atrial fibrillation	Yes	1

*ASPECTS, Alberta Stroke Program Early Computed Tomography Score; CNS, Canadian Neurological Scale; CT, computed tomography; GCS, Glasgow Coma Scale; IV-tPA, intravenous tissue plasminogen activator; LACS, lacunar stroke syndrome; mRS, modified Rankin Scale; NIHSS, National Institutes of Health Stroke Scale; OCSP, Oxfordshire Community Stroke Project; PACS, partial anterior circulation stroke syndrome; POCS, posterior circulation stroke syndrome; TACS, total anterior circulation stroke syndrome; TIA, transient ischemic attack; TICI, Thrombolysis In Cerebral Ischemia; TOAST, Trial of ORG 10172 in Acute Stroke Treatment*.

* *Risk score was not developed*.

** *Optional variable*.

For seven scales the outcome of interest was a specified range of scores on the modified Rankin Scale (mRS) (34–36). The scale is a measure of functional outcome following stroke, ranging from no symptoms (a score of zero) through increasing levels of disability, to death (a score of six). Five scales focused on mortality, while one scale aimed to predict recurrent stroke and another length of hospital stay. Four scales predicted more than one outcome.

Our critical appraisal of scales' development process identified potential sources of bias in each study, as well as issues related to incompleteness of reporting for methods and results. Most common limitations were around handling missing data and model development. In relation to the former, in two studies it was not clearly stated how missing data was handled. Six studies used complete-case analysis, and the remaining two excluded participants from analyses involving the particular variables they had no data for. For model development, six studies selected variables for multivariable modeling based on the univariable significance (Table 5).

**Table 5:** Assessment of risk of bias in scale derivation studies.

**Scale**	**Data source**	**Participants**	**Outcome**	**Predictors**	**Sample size**	**Missing data**	**Model development**	**Model performance**	**Model evaluation**	**Results**
Astral										
Dragon										
FSV										
iScore										
PLAN										
SNARL										
SOAR										
SPI										
S-TPI										
THRIVE										

*Color code: green, low risk of bias; yellow, unclear or medium risk of bias; red, high risk of bias*.

We present an overview of each scale, focusing on scale content, development, validation, and where applicable any modification to the scale. We summarize our critical appraisal of derivation studies and discuss potential issues around implementation of the scales in routine clinical practice.

### Acute Stroke Registry and Analysis of Lausanne (ASTRAL)

#### Scale Content and Development

The ASTRAL scale uses six input variables to predict unfavorable functional outcome at 3 months (mRS>2): age, stroke severity according to the National Institutes of Health Stroke Scale (NIHSS) (37), time from symptom onset to admission, range of visual fields, acute glucose, and level of consciousness (38). Based on these variables, an integer score is assigned, from zero with no upper limit. Higher scores are associated with a greater probability of an unfavorable outcome. Through a logistic regression procedure, the scale was developed in a sample of 1,633 ischemic stroke patients from the Acute Stroke Registry and Analysis of Lausanne (39).

#### Scale Validation and Updating

Using a 2-fold cross-validation technique for internal validation, the scale was found to have good discriminatory power, AUROC:0.85 for prediction of mRS>2 at 3 months. The derivation paper further described external validation of the scale in two independent cohorts from Athens and Vienna (40, 41), reporting AUROC values of 0.94 and 0.77, respectively. Calibration was assessed in all three cohorts based on Hosmer-Lemeshow test and inspection of calibration plots, indicating a good fit with the data.

The ASTRAL scale has been subsequently externally validated by seven studies, with six assessing predictive value based on AUROC estimates (42–48). Within these, ASTRAL was found to have fair to good discriminatory power, with the exception of one study, involving a Brazilian cohort (AUROC 0.67) (44). These external validation studies used differing time points for outcome assessment (up to 5 years post-stroke) and differing outcomes, including mortality and symptomatic intracerebral hemorrhage (sICH).

#### Critical Appraisal and Clinical Application

In the ASTRAL derivation study we found potential sources of bias relating to participant selection, namely excluding all patients with pre-stroke dependency and any missing data. In addition, treatment effects were not accounted for. We also noted that some issues relevant to scale development were unclear: whether any method of blinding was used, the number of candidate predictors (which allows to estimate whether the sample size was sufficient), and finally whether there were any significant baseline differences between the derivation and validation cohorts.

Despite these concerns, evidence from validation studies suggests that the predictive performance of ASTRAL is sufficient for the clinical setting. The scale was designed with the acute context in mind, and does not require sophisticated diagnostic tests. Nonetheless, in some cases, estimating onset to admission time may not be possible. Where all necessary information is accessible, the ASTRAL offers an easily-calculable score, with use aided by color-coded graphs to assign a percentage probability of unfavorable outcome based on clinical features. There is also a score calculator available online (49).

### Dense Artery, mRS, Age, Glucose, Onset-to-Treatment, and NIHSS (DRAGON)

#### Scale Content and Development

The DRAGON scale incorporates the six variables in its acronym, as well as early infarct signs on computed tomography (CT). It was developed to predict functional outcome at 3 months in stroke patients treated with intravenous tissue plasminogen activator (IV-tPA) (50). The outcome was trichotomized according to mRS scores, where mRS 0–2 was defined as “good outcome,” mRS 3–4 as “poor outcome” and mRS 5–6 as “miserable outcome.” Scale scores range from one to ten, with higher values associated with poorer outcomes. The scale was derived in a single-center Finish cohort of 1,319 ischemic stroke patients, using a logistic regression procedure.

#### Scale Validation and Updating

On internal validation, using 1,000 bootstrap replications, DRAGON was found to have an AUROC of 0.84 (95%CI: 0.80–0.87) for prediction of miserable outcome. A comparable AUROC value was obtained through external validation, performed by the authors in a cohort of 330 Swiss patients: 0.80 (95%CI: 0.74–0.86). Calibration was not assessed. DRAGON has undergone subsequent external validation in ten studies (42, 43, 46, 51–57), all of which concluded the scale performs well, and (where assessed) had fair to good discriminatory power. In majority of cases, the scale was used in a similar context and for the same purpose as in the derivation study. However, one study assessed prediction of sICH (42).

Recognizing the increasing use of magnetic resonance imaging (MRI), the original DRAGON scale was adapted to include MRI based variables (58). Namely, with all clinical variables remaining unchanged, proximal middle cerebral artery occlusion on MR angiography replaced hyperdense artery sign, and the diffusion-weighted imaging Alberta Stroke Program Early Computed Tomography Score (DWI ASPECTS) replaced CT early infarct signs (59, 60). The scale was derived in a French cohort of 228 patients treated with IV-tPA. Internal validation was performed using a bootstrapping method. For prediction of 3-month mRS>2, MRI-DRAGON was found to have an AUROC of 0.83 (95%CI: 0.78–0.88). The scale was externally validated in one subsequent study, where reported AUROC values for prediction of poor and miserable outcome were 0.81 (95%CI: 0.75–0.87) and 0.89 (95%CI: 0.84–0.95), respectively (61).

#### Critical Appraisal and Clinical Application

We identified issues in the DRAGON derivation study. All continuous candidate predictors were categorized. A complete-case analysis approach was employed and discriminatory power was only estimated for prediction of miserable outcome, while calibration was not assessed at all. Moreover, it seemed unclear whether any blinding method was applied for assigning mRS scores, and there were no description of the multivariable method for selection of final predictors.

In the context of clinical practice, DRAGON score should be easy to calculate [online score calculator available (62)]. Again, estimating symptom onset-to-treatment time may not be possible in some cases. There is potential for misinterpretation of early infarct and hyperdense cerebral artery signs (63–65). From this point of view, MRI-DRAGON appears a valuable alternative. MRI has been found to be a more sensitive method for ischemia detection than CT, and use of a semi-quantitative assessment of lesions is likely to ensure higher reproducibility (66). Importantly, based on results of validation studies, both versions of the scale seem to have satisfactory predictive ability, although evidence on performance of MRI-DRAGON is still limited.

### Five Simple Variables (FSV)

#### Scale Content and Development

The FSV scale incorporates two models for predicting functional outcome at 6 months post-stroke (67–69). One is used for good (mRS<3) or excellent (mRS<2) outcomes, and one for prediction of a devastating outcome (mRS>4; FSV_DEV_). The two models share four input variables: age, pre-stroke functional status (Oxford Handicap Score) (70), ability to lift both arms off the bed, and normal verbal response on the Glasgow Coma Scale (71). The first model additionally includes ability to walk unaided, while the FSV_DEV_ incorporates stroke subtype. Prediction scores created based on the models range from−5 to 5 for the positive outcomes, and 0 to 15 for the devastating outcome. In both cases, a higher score is associated with a greater likelihood of having the outcome of interest. Both FSV models were derived in a single-center Canadian cohort of 538 stroke patients.

#### Scale Validation and Updating

Internal validation of the prediction scores, using 500 bootstrap replications, indicated good discriminatory power, with AUROC values of 0.88, 0.87, and 0.86 for good, excellent and devastating outcomes, respectively. Similar results were reported for initial external validation, conducted in a sample of patients from the Oxfordshire Community Stroke Project (OCSP), with AUROC values ranging from 0.86 to 0.89. Calibration was assessed only in the derivation sample for prediction of good outcome, and, based plotted calibration curves, concluded to be good (67).

FSV scores have been externally validated in one study (72), reporting good discriminatory power for prediction of good and devastating outcomes at 6 months in a Scottish stroke cohort. The use of five variables for predicting post-stroke functional outcome was also assessed in a cohort combining six European populations. However, here a similar scale was being independently developed rather than the FSV being externally validated (73). The described model included the same variables, although a different measure was used for estimating pre-stroke functional status [Barthel index (74)]. The authors reported good discriminatory power on both internal and external validation.

#### Critical Appraisal and Clinical Application

To assess FSV derivation, we reviewed three publications and identified potential sources of bias. The sample size was insufficient for the number of tested candidate predictors. Although a complete-case analysis method was not applied, with no data imputation, participants with missing data were excluded from particular analyses. Blinding was unclear. Input variables for multivariable analyses were selected based on univariate significance. In the paper where models for excellent and devastating outcomes were developed, calibration was not assessed, while in the remaining two, the procedure was mentioned but no calibration plots were presented. In relation to study results, differences in baseline characteristics between derivation and validation datasets were not assessed, and a data-driven approach was applied when selecting cut-off scores for outcome prediction (75).

In clinical practice, a significant advantage of FSV is the use of easily accessible and often routinely collected information. Moreover, for patients and their families, the differentiation between recovering to a level of functional independence with and without disability can be of particular value. It is unlikely however for this useful concept to be transferred into practice, as the same FSV cut-off score was chosen for both outcomes, the difference lying in prognostic accuracy for prediction of each. Finally, although reports on FSV performance are encouraging, further external validation studies are necessary before it can be considered for use in a clinical setting.

### iScore

#### Scale Content and Development

The iScore was developed using a logistic regression procedure to predict death at two timepoints. The derivation study included 12,262 ischemic stroke patients from the Registry of the Canadian Stroke Network (76). For outcome prediction at 3 months, an integer score (from zero, with no defined upper limit) is calculated based on: age, sex, stroke severity assessed with the Canadian Neurological Scale (77), stroke subtype according to the Trial of ORG 10172 in Acute Stroke Treatment (TOAST) (78), acute glucose, history of atrial fibrillation, congestive heart failure, cancer, kidney disease, and preadmission dependency. For predicting one-year mortality, previous myocardial infarction and smoking status are added. Higher scores associated with greater mortality.

#### Scale Validation and Updating

In the derivation study, a split-sample validation method was chosen, with 8223 patients assigned to the development set (AUROC: 0.85 and 0.82 for 30-day and 1-year mortality, respectively) and 4039 in the internal validation (AUROC: 0.85 and 0.84). External validation used data from 3270 patients from the Ontario Stroke Audit. AUROC: 0.79 and 0.78, for 30-day and 1-year mortality, respectively.

The scale has been further externally validated in 15 studies (48, 54, 79–91). The iScore has been applied not only to predict mortality, but also poor functional outcome, institutionalization, clinical response, hemorrhagic transformations following thrombolytic therapy, and healthcare costs. All studies concluded that iScore is useful, predicting outcomes of interest with sufficient accuracy. Where AUROC values were estimated, they were fair to good, apart from one study where AUROC was 0.68 for 30-day mortality or disability at discharge (79).

Recognizing the difficulty of etiological classification (92), a revised iScore (iScore-r) was developed, replacing TOAST with OCSP (93). The revised scale was validated in a Taiwanese cohort of 3,504 ischemic stroke patients, for prediction of poor functional outcome (mRS>2) at discharge and at 3-months. Assessment of discriminatory power in an external cohort of iScore and iScore-r indicated comparable performance of the scales. AUROC of 0.78 and 0.77 for discharge outcome, and AUROC of 0.81 and 0.80 for 3-month outcome, with lower values reported for iScore-r.

#### Critical Appraisal and Clinical Application

We identified limitations in the iScore derivation. A complete-case analysis approach was applied. Variables were selected based on univariable significance. Administration of treatments was not accounted for. A split-sample method was used for internal validation, while the external validation cohort was partially recruited from the same centers as the derivation cohort, which gives overoptimistic estimates of performance in independent populations. It was unclear whether blinding was applied; which inputs were included in the model as continuous and which were categorized; and how pre-stroke dementia (a candidate predictor) and dependency were operationalized.

The iScore scale has many external validation studies, which indicate sufficient prognostic ability for outcomes other than just mortality. Use of the scale can be aided by an online score calculator (94). Nonetheless, compared to most scales included in this review iScore require substantial baseline information. The revised scale may offer a solution to the issues of acute classification, yet the iScore-r derivation study reported high attrition rates, and with no further external validation studies, the generalizability of the scale remains uncertain.

### Preadmission Comorbidities, Level of Consciousness, Age, and Neurological Deficit (PLAN)

#### Scale Content and Development

The PLAN scale was developed to estimate probability of death and severe disability following ischemic stroke, specifically 30-day and 1-year mortality, and mRS>4 at discharge (95). A risk score ranging from 0-25 is calculated based on: pre-admission dependency, history of cancer, congestive heart failure, atrial fibrillation, consciousness, age, proximal weakness of the leg, weakness of the arm, aphasia and neglect. Higher scores are associated with greater likelihood of death or severe disability. The scale was derived through logistic regression using the same multicenter data source as in the case of iScore. The baseline sample comprised 9,847 patients. However, as a split-sample validation method was applied, only 4,943 of subjects were included in the development set.

#### Scale Validation and Updating

The derivation study reported results of both apparent and internal validation, with AUROC values ranging from 0.82 to 0.89 for all three outcomes. The scale's performance was not assessed in an independent dataset. External validation was however conducted in two subsequent studies (48, 73). The scale was applied for prediction of good functional outcome, poor outcome, and mortality. In all analyses, PLAN was found to have AUROC values above 0.80.

#### Critical Appraisal and Clinical Application

Our assessment of PLAN revealed issues predominantly related to three aspects of scale development: predictors, the model derivation procedure, and assessment of performance. In relation to predictors, all originally continuous variables were categorized. There was also a lack of reporting on how pre-stroke dementia and dependency were operationalized, as well as on blinding to outcome for assessment of input variables. In terms of creating the model, variables for multivariable analysis were chosen based on estimated associations in univariable analysis, while the method for selecting final predictors in multivariable analysis seemed unclear. Finally, the scale was only internally validated, using a split-sample method. Calibration was assessed alongside discrimination, however this was limited to performing the Hosmer-Lemeshow test and correlations between observed and expected outcomes. An additional concern is the lack of statement on the method of handling missing data.

Given the increasing use of IV-tPA as a treatment option in ischemic stroke, it is noteworthy that patients receiving this intervention were excluded from the PLAN derivation study. This does not necessarily entail limited applicability of the scale, particularly as the external validation studies, reporting good performance for PLAN, both included IV-tPA-treated patients. However, as the scale was only applied in two independent dataset, it seems that more evidence is necessary before reaching conclusions on PLAN's generalizability. If an acceptable level of performance is consistently indicated, another issue worth investigating will be whether the relative complexity in scoring impedes implementation of the scale in clinical practice.

### Symptomatic Hemorrhage, Baseline NIHSS, Age, Reperfusion, and Location of Clot (SNARL)

#### Scale Content and Development

The SNARL scale uses the three clinical and two imaging variables in its acronym to predict a good outcome (mRS < 3) at 3 months following ischemic stroke treated with endovascular therapy (96). Scores can range from zero to eleven, with higher scores associated with a greater probability of a good outcome. The scale was derived through a logistic regression procedure, using data of 511 patients from a multicenter registry.

#### Scale Validation and Updating

Based on results of apparent validation, reported AUROC was 0.79 (95%CI: 0.75–0.83). The study also assessed the scale's performance in an independent cohort, comprising 223 patients from the North American Solitaire Acute Stroke registry. For this dataset, AUROC was 0.74 (95%CI: 0.68–0.81). In addition, the authors reported that compared to the THRIVE scale (described below), SNARL presented a 35% improvement in terms of accurately classifying patients' probability of a good outcome. We did not identify any further external validation studies assessing this scale.

#### Critical Appraisal and Clinical Application

Through our critical appraisal of the SNARL derivation study we identified two sources of bias, both common across the reviewed scales, use of a complete-case analysis approach and selection of predictors based on associations in a univariable statistical procedure. The applied input selection process in multivariable analysis, on the other hand, seemed unclear, as did the use of any blinding methods. Finally, although predictors were well-operationalized, interpretation of imaging findings may be subject to relatively high interobserver variability.

### Stroke Subtype, OCSP, Age, and Pre-stroke mRS (SOAR)

#### Scale Content and Development

SOAR was developed to predict early mortality (inpatient and 7-day) and length of hospital stay, based on the four clinical variables of the scale's acronym (97). Using a logistic regression model, a scoring system ranging from 0 to 8 was derived, with higher scores associated with a greater likelihood of death and extended length of stay. The derivation cohort included 12,355 acute stroke patients (91% ischemic) from a multicenter register, based in the United Kingdom.

#### Scale Validation and Updating

SOAR was internally validated using a bootstrapping resampling method, with reported AUROC values being the same for both 7-day and inpatient mortality: 0.79 (95%CI: 0.78–0.80). For predicting length of hospital stay, dichotomized at seven days, AUROC was 0.61 (95%CI: 0.60–0.62). Although external validation was not included as part of the derivation paper, SOAR has been subsequently assessed in independent datasets in five studies (98–103). Four studies assessed the scale's performance for predicting early mortality (inpatient, 7-day, discharge, and 90-day). Three found SOAR to have fair discriminatory power, and one, good. One study applied the scale for prediction of length of hospital stay. Discrimination was not formally assessed, however the authors reported that SOAR scores were significantly associated with the outcome (100).

Three external validation studies additionally aimed to improve the scales predictive performance by adding new variables. The modified SOAR (mSOAR) added stroke severity (NIHSS) (98). When compared to SOAR, mSOAR was found to have superior discriminatory power: AUROC of 0.83 (95%CI: 0.79–0.86) vs. 0.79 (95%CI: 0.75–0.84). Noteworthy, performance was assessed in the mSOAR derivation set. Nonetheless, the finding was confirmed in an independent Chinese patient sample: AUROC of 0.78 (95%CI: 0.76–0.81) and 0.79 (95%CI: 0.77–0.80) for discharge and 90-day mortality, respectively, compared to 0.72 (95%CI: 0.70–0.75) and 0.70 (95%CI: 0.69–0.72), respectively, with higher estimates reported for mSOAR. The remaining two updates of SOAR included adding admission blood glucose levels (SOAR-G) and admission sodium (SOAR-Na) (101, 103). Both concluded that the original and revised scales performed well, however without evidence of the latter offering a significant improvement in discriminatory power.

#### Critical Appraisal and Clinical Application

Reviewing the SOAR derivation study, we noted that the authors intended to select predictors for multivariable analysis based on univariable associations. However, as all candidate predictors were found to be significantly associated with the outcome, using this approach would not have influenced the results. In this case, what seems to be a greater issue, is that sex was not included in the final model, despite the significance of its association in both univariable and multivariable analyses. Risk of bias was increased by excluding all patient with missing data from the study, as well as by not accounting for effects of administered treatments.

For implementation in clinical practice, the simplicity of SOAR appears a major advantage, including easily accessible information on only four variables. Adding NIHSS is the only attempted modification that has significantly improved scale performance. In many centers, where the measure is not routinely used, this will introduce an additional challenge, yet it is worth considering that stroke severity has been consistently found to be associated with post-stroke outcomes. Calculation of mSOAR can be aided by use of an online tool (104).

### Stroke Prognosis Instrument (SPI)

#### Scale Content and Development

SPI was developed to predict risk of stroke or death within 2 years of TIA or minor stroke (105). A score ranging from 0 to 11 is calculated based on five variables: age, history of diabetes and coronary heart disease, acute hypertension, and presentation (TIA or minor stroke). This score assigns patients to one of three risk groups: low (0–2 points), medium (3–6 points), and high (7–11 points). The scale was developed based on survival analysis, specifically using a Cox proportional hazards model. The derivation cohort included 142 patients, who had undergone carotid ultrasonography in a United States tertiary care hospital. Based on data from this sample, an initial SPI score was developed, including only three variables: age, diabetes, and hypertension.

#### Scale Validation and Updating

In the derivation study, the SPI score was assessed based on its ability to accurately stratify patients according to risk of stroke or death, using data from the development sample, as well as in an independent Canadian cohort, including 330 patients. In the derivation set, the results showed that 3% of patients estimated as being at low risk had a subsequent stroke or died within 2 years of the initial neurovascular event, while the incidence for patients assigned to the medium risk group was 27%, and for those in the high-risk group 48%. For the validation cohort, the incidence of stroke and death were 10, 21, and 59%, for the 3 risk groups respectively. To ameliorate decreased performance estimates in the external set, two more variables were added to the scale, differentiation between a TIA and a minor stroke, and a history of coronary heart disease.

The authors of SPI subsequently externally validated the final scale in four independent cohorts, and used one of these cohorts to develop a modified version of the scale (106). SPI-II was derived based on data from 525 female patients, who participated in the Women's Estrogen for Stroke Trial (107). In addition to the original variables, SPI-II incorporates history of congestive heart failure and prior stroke, with total scores ranging from 0 to 15. Data from three cohorts, with a total of 9,220 patients, were used in a pooled analysis to estimate the AUROC values for both scales, concluding that SPI-II (0.63; 95%CI: 0.62–0.65) had superior discriminatory power to SPI-I (0.59; 95%CI: 0.57–0.60).

SPI-II has been subsequently externally validated in two studies (108, 109). The first found that for prediction of both stroke and death at 1 year, SPI-II had poor discriminatory power (0.62; 95%CI: 0.61–0.64), which further decreased when limiting the outcome measure to recurrent stroke (0.55; 95%CI: 0.51–0.59). In the second study, groups identified as medium and high risk were combined, and the scale applied to predict 3-month recurrence of ischemic events. Here, the scale was found to have an AUROC of 0.55 (95%CI: 0.41–0.69).

#### Critical Appraisal and Clinical Application

The SPI derivation study had a high risk of bias. Exclusion criteria for study participation included previous stroke and any missing data on variables of interest. As a result, close to 60% of the baseline sample were not included in the analyses, leaving an insufficient number of participants relative to the number of predictors that were investigated. All of these predictors were categorized. Distinguishing between TIAs, minor strokes, and stroke has potential for interobserver variability. All candidate predictors were included in analysis, however forward selection method was used.

In relation to assessing scale performance, we noted that neither discrimination nor calibration were assessed in the SPI derivation study. The chosen validation cohort also differed from the derivation set in that some predictors were measured in alternative ways, and patients with previous strokes were included. The latter introduced an additional problem, as a history of cerebrovascular events was found to be significantly associated with the outcome. However, as this could not be investigated in the derivation set, the variable was not incorporated as a predictor. The he final SPI score seemed to be derived on the basis of a partially erroneous process of rounding up variable coefficient values.

SPI-II is also at high risk of bias, using data from a female-only patient sample. Although the revised scale was found to have significantly increased discriminatory power compared to the original, it was nonetheless poor, as confirmed in subsequent validation studies. The scale's predicted outcome is also problematic, creating a highly heterogenous risk group. On one hand, with a highly diverse range of possible scenarios, identifying a set of predictors both necessary and sufficient for accurate outcome prognosis seems extremely difficult. On the other hand, for clinicians, and particularly for patients, identifying that one belongs to a high-risk group seems of limited value, when this can indicate increased likelihood of anything from a minor stroke with no residual disability to death.

### Stroke Thrombolytic Predictive Instrument (S-TPI)

#### Scale Content and Development

S-TPI was developed to assist clinicians in predicting the outcome of ischemic stroke patients following intravenous IV-tPA (110). Two logistic regression models were created: one for prediction of good outcome (mRS<2) and one for prediction of catastrophic outcome (mRS>4), at 3 months. In addition to IV-tPA treatment, the former model included the following variables: age, initial systolic blood pressure, diabetes, sex, baseline NIHSS score, prior stroke, and symptom onset to treatment time; as well as interaction terms: treatment with blood pressure, sex, prior stroke, and onset to treatment time, and age with NIHSS. For prediction of catastrophic outcome, the model consisted of considerably fewer inputs: age, NIHSS, serum glucose and ASPECTS score, the latter treated as an optional variable, with inclusion subject to availability. The models were derived using a combined dataset from five randomized clinical trials of IV-tPA, involving 1983, 1967, and 1883 patients (depending on the model), out of an initial cohort of 2184.

#### Scale Validation and Updating

The models were internally validated using a bootstrapping method, creating development and independent test datasets. In the latter, AUROC values were 0.77 [interquartile range (IQR): 0.76–0.78] and 0.76 (IQR: 0.75–0.78), for prediction of good outcome and catastrophic outcome without ASPECTS, respectively. Calibration was graphically assessed through plotting mean predicted vs. observed rates of patient outcomes across quintiles, and was concluded to be excellent.

S-TPI was subsequently externally validated in three studies (111–113). Two studies assessed discriminatory power based on AUROC, finding the scale to have fair to good performance for both good and catastrophic outcomes. Calibration curves were investigated in all three studies. In each case, S-TPI was found to overestimate the likelihood of a good outcome, particularly at higher levels of observed probabilities. In relation to a catastrophic outcome, findings were mixed, two studies reported the scale to underestimate the likelihood of this outcome, while the third concluded the opposite. One of the studies undertook recalibration of the scale and further added two variables for prediction of good outcome, signs of infarction on brain scan and serum glucose level. The authors reported this improved the scales discriminatory power (AUROC of 0.77 vs. 0.75) (112).

In contrast, the group that developed S-TPI sought to simplify the scale by reducing the number of predictors, with an aim to makes its implementation in routine clinical practice more feasible (114). The process involved removing interaction terms with limited external supporting evidence, removing the ASPECT score, and exploring the use of simpler stroke severity measures. A total of nine models were generated through logistic regression, for prediction of three outcome levels: mRS<2, mRS<3, and mRS>4. Results from apparent validation showed that AUROC values for all models ranged from 0.75 to 0.80. External validation was performed for models predicting mRS<2 and mRS<3, with findings indicating comparable discriminatory power as in the derivation set. The authors concluded that reducing model components did not lead to a substantial deterioration in performance. We have not identified any further publications externally validating the simplified S-TPI models.

#### Critical Appraisal and Clinical Application

Risk of bias in the derivation paper was increased by use of data from randomized control trials. Inclusion and exclusion criteria for such trials typically lead to recruiting a highly selective group of participants, thus decreasing the generalizability of scales developed based on their data. In addition, it seemed that trial investigators were not blinded to predictors (with the exception of use of IV-tPA vs. placebo) when assessing the outcome. Finally, although patients with missing data were not excluded from the derivation study outright, lack of data imputation would have led to participants being excluded from particular analyses, when they had no data for one or more of the variables used.

In view of scale implementation, it is important to note that individual patient outcome predictions were to be estimated automatically using a computer system, with an open-access version of the instrument also published online. The latter is however no longer available. With no presentation of an easily calculable risk score, estimating probabilities of patient outcomes would be a challenging task for clinicians, particularly taking into account the complexity of the S-TPI models. Despite the effort to simplify the scale, its use would nonetheless require applying the regression model formulae itself. There also seems to be no clear indication of which version of the multiple S-TPI models is the best candidate for implementation. Overall, it appears more evidence of predictive performance is needed before the simplified models can be considered for clinical use, as well as an easily-applicable scoring system.

### Totaled Health Risks in Vascular Events Stroke (THRIVE)

#### Scale Content and Development

The THRIVE scale was originally developed with an aim to support identification of patients who may benefit from endovascular stroke treatments, in terms of 3-month functional outcome and risk of death (115). The scale includes five clinical variables: age, stroke severity, and history of hypertension, diabetes mellitus and atrial fibrillation. On their basis, an integer score is calculated ranging from 0 to 9, with higher scores associated with a greater probability of a poor outcome. The scale was developed using logistic and ordinal regression models. The derivation cohort included participants of the MERCI and Multi MERCI trials of mechanical thrombectomy, with a total of 305 ischemic stroke patients (116, 117).

#### Scale Validation and Updating

The derivation paper reported results of apparent validation for prediction of good outcome (mRS < 3), finding that the final prognostic model had an AUROC of 0.71. The THRIVE score, developed based on estimated odds ratios for each predictor, was assessed in terms of its association with the percentage of patients with a particular outcome. A good outcome was observed in 64.7% of patients with a score of 0–2 and in 10.6% of cases with a score of 6–9. Reported mortality rates were 5.9 and 56.4% for patients with low and high THRIVE scores, respectively.

There have been 16 subsequent studies externally validating THRIVE. These involved patient groups receiving intra-arterial therapy, intravenous thrombolysis, and no acute treatments, and focused on a number of different outcomes – good functional outcome, poor outcome, risk of hemorrhagic transformations, infarct size, and even pulmonary infection (44, 118–132). The majority of studies aimed to predict multiple outcomes, and 15 assessed the scales predictive performance in terms of AUROC values, typically alongside other estimates. Seven studies found the discriminatory power to be either poor or fair, depending on the outcome, four reported it to be poor for all used outcomes, three to be fair, and only one study found the performance to be good, specifically for prediction of mortality rates.

With an aim to improve the scale's predictive performance, a revised version was developed, the THRIVE-c Calculation (133). The modified tool includes the same variables as the original scale, with age and NIHSS score entered as continuous rather than categorized variables. The derivation study reported results of apparent, internal (split-sample) and external validation, with AUROC values ranging from 0.77 to 0.80 for prediction of poor outcome. In the overall study cohort, THRIVE-c was found to have significantly superior predictive performance compared to the original THRIVE score (0.79, 95%CI: 0.78–0.79 vs. 0.75, 95%CI: 0.74–0.76). THRIVE-c has been subsequently externally validated in a Chinese population of patients receiving IV-tPA (134). The scale was used to predict symptomatic hemorrhage, poor functional outcome and mortality, with reported AUROC values of 0.70, 0.75, and 0.81, respectively.

#### Critical Appraisal and Clinical Application

Our critical appraisal of the THRIVE derivation study indicated issues with each of the assessed aspects, either due to methodological quality or incomplete reporting. The derivation set consisted of participants recruited to a clinical trial, thus leading to participation of a selective group of subjects. Moreover, the final sample size and method of handling missing data seemed unclear. In relation to input variables, the initial set of candidate predictors appeared limited, omitting a number of factors found to be associated with functional outcome in previous research. Inclusion of specific chronic diseases in multivariable analysis was based on significance of associations in univariable analysis. Three factors, age, stroke severity and success of vessel recanalization, were included in multivariable analyses outright, and all were found to be independently associated with the outcome. It is however unclear why vessel recanalization was not incorporated into the final THRIVE scale. There was also no report of how cut-offs were determined for the derived THRIVE score.

Assessment of scale performance in the derivation study was limited to apparent validation. Moreover, discriminatory power was tested only for the model predicting good outcome; it was not assessed for the model predicting mortality or for the derived THRIVE score. Although the scale has undergone extensive external validation since its development, findings from these studies do not seem to support a favorable judgement on the scale's prognostic performance. THRIVE-c appears to be a superior alternative, yet up-to-date we have found only one independent validation study assessing the scale's predictive ability. In view of use in routine clinical practice, inclusion of relatively few variables, based on information typically available in an acute setting, is a relevant advantage of THRIVE. Score calculation can additionally be aided be use of an online tool (135). However, existing evidence on predictive ability does not seem to merit implementation.

## Discussion

There are many prognostic tools available for use in acute stroke settings. We have reviewed a selection of these and common themes emerge. Our primary interests were methodological quality of derivation, subsequent external validation and scale usability in routine clinical practice. Across 10 primary derivation studies of better-known scales, we identified potential sources of bias in each. However, it is the results of external validation studies that allow us to conclude on the scales prognostic value and applicability. We found that all scales, but one, were externally validated.

While there was a range of prognostic accuracies reported, most scales had properties that would be considered “acceptable.” This is perhaps not surprising as the scales tended to measure the same concepts of demographics, comorbidity, initial stroke severity and pre-stroke functional status. Where scale developers have tried to add additional elements to these core predictors, the gain in predictive power has been limited. However, most scales have been developed with a biomedical focus and it is plausible that other less traditional factors could improve utility of the scales, for example incorporating measures of frailty, resilience, provision of rehabilitation services and social support, or the clinician's clinical gestalt.

Based on our literature search, we identified the highest number of external validation studies for THRIVE. Yet results indicated a level of predictive performance insufficient to merit the scale's use in a clinical setting. Our critical appraisal may partly explain this, identifying concerns relating to all aspects of THRIVE's derivation process. Four other scales, ASTRAL, DRAGON, iScore, and SOAR, have also been validated in multiple independent datasets. For all, findings suggested a level of predictive ability that would merit implementing the scales in clinical practice. Moreover, one study reported ASTRAL and DRAGON to predict patient outcomes more accurately than clinicians (46). Although evidence regarding the performance of other scales included in this review seemed insufficient to reach firm conclusions, a number of these tools were derived with relatively low risk of bias, and future research is likely to confirm their prognostic value. These include PLAN, SNARL, S-TPI, as well as updated scale versions: MRI-DRAGON, iScore-r and mSOAR.

For a number of reasons, it is challenging to directly compare the reviewed prognostic tools, and we have deliberately chosen to avoid naming a single preferred tool. Firstly, studies assessing more than one scale as part of an external validation process are relatively uncommon. When conducted, findings are often difficult to interpret, small differences in predictive ability between scales may arise from the superiority of one over another, but they could also be attributed to an incidentally greater similarity between the validation set and the derivation set of the scale found to perform best. Secondly, although satisfactory predictive ability is essential for a scale to be clinically useful, it is not sufficient. A number of other factors need to be considered, including feasibility in routine clinical practice, the relevance of the predicted outcome to the specific context, and whether applying the tool improves clinical decision-making, patient outcomes or cost-effectiveness of services (136). To help answer these questions, it is necessary to conduct impact studies, a stage in prognostic research that to our knowledge none of the described scales have yet reached.

Our focused literature review has strengths and limitations. We recognize that there have been many high quality systematic and narrative reviews of stroke prognosis scales (10, 137). We hope that our review offers a novel focus. We have appraised relevant stroke scales against each other; very few derivation papers have done this, despite its importance when choosing which scale to use. Additionally, we have followed the PRISMA systematic review guidelines (9) when designing and completing our study and based our appraisal on the CHARMS checklist (16). Our intention was not to offer a comprehensive review, rather we choose exemplar scales that featured in high impact journals and so by implication would be amongst the best known in the clinical community. In our assessment of feasibility we identified clinical and radiological features that may be challenging to assess in the acute setting (63, 138). Our focus was routine stroke practice (139) and our comments on feasibility may not apply to specialist stroke centers. It takes time for scales to become established and our review did not include recently published scales, for example those designed to inform thrombectomy decisions (140). However, the literature describing these scales is increasing rapidly and soon there may be sufficient validation studies.

We used data from our focused literature review to compare long-term stroke prognosis scales. We found many scales with similar content and properties. Although development of the scales did not always follow methodological best practice, most of these scales have been subsequently validated. Rather than developing new scales, prognostic research in stroke should now focus on implementation and comparative analyses.

## Author Contributions

SS and TQ contributed to the conception and design of the study. TQ designed the literature search strategy, SS performed the search and screened the results. SS and BD extracted study data, all authors were involved in critical appraisal of included studies. BD and SS drafted the final manuscript. All authors critically revised the manuscript, approved its final version, and agreed to be accountable for its content.

### Conflict of Interest Statement

The authors declare that the research was conducted in the absence of any commercial or financial relationships that could be construed as a potential conflict of interest.
