# Supplementary material for: Thinking About the Future: A Review of Prognostic Scales Used in Acute Stroke
Source: Front Neurol. 2019 Mar 21;10:274. doi: 10.3389/fneur.2019.00274 (PMC6437031; doi:10.3389/fneur.2019.00274)
Supplement: Supplementary file 1 [file Table_1.DOCX]

Supplementary Material

Thinking about the future - A review of prognostic scales used in acute stroke.

# Literature search strategy

## Inception to October 2015

1 (cerebrovascular disorders or basal ganglia cerebrovascular disease or brain ischemia or carotid artery diseases or intracranial arterial diseases or intracranial arteriovenous malformations or "intracranial embolism and thrombosis" or intracranial hemorrhages or stroke or brain infarction or vasospasm, intracranial or vertebral artery dissection or (stroke or poststroke or post-stroke or cerebrovasc$ or brain vasc$ or cerebral vasc$ or cva$ or apoplex$ or SAH) or ((brain$ or cerebr$ or cerebell$ or intracran$ or intracerebral) adj5 (isch?emi$ or infarct$ or thrombo$ or emboli$ or occlus$)) or ((brain$ or cerebr$ or cerebell$ or intracerebral or intracranial or subarachnoid) adj5 (haemorrhage$ or hemorrhage$ or haematoma$ or hematoma$ or bleed$)) or (hemiplegia or paresis) or (hemipleg$ or hemipar$ or paresis or paretic)).kw,ti,ab. (683556)

2 (prognos* or predict*).ti. or ((scor* or risk* or predict* or prognostic*) adj1 (system* or model* or equation* or rule* or tool* or scor*)).ti,kw. or (multivaria* adj1 (scor* or model* or equation* or tool* or rule*)).ti,kw. or "receiver operating characteristic*".ti,kw,ab. or c?statistic.ti,ab,kw. or calibrat*.ti,kw. or validat*.kw,ti. or "roc curve/".kw,ti,ab. (890752)

3 (stroke or "cerebrovascular diseases" or "age and ageing" or "journal of the american geriatrics society" or neurology or "lancet neurology" or "archives of neurology" or lancet or JAMA or BMJ or "new england journal of medicine").jn. (704474)

4 1 and 2 and 3 (5511)

5 limit 4 to english language (5510)

6 limit 5 to full text (4056)

7 limit 6 to human [Limit not valid in HAPI; records were retained] (3636)

8 limit 7 to yr="2005 -Current" (2482)

## October 2015 to May 2018

1 (cerebrovascular disorders or basal ganglia cerebrovascular disease or brain ischemia or carotid artery diseases or intracranial arterial diseases or intracranial arteriovenous malformations or "intracranial embolism and thrombosis" or intracranial hemorrhages or stroke or brain infarction or vasospasm, intracranial or vertebral artery dissection or (stroke or poststroke or post-stroke or cerebrovasc$ or brain vasc$ or cerebral vasc$ or cva$ or apoplex$ or SAH) or ((brain$ or cerebr$ or cerebell$ or intracran$ or intracerebral) adj5 (isch?emi$ or infarct$ or thrombo$ or emboli$ or occlus$)) or ((brain$ or cerebr$ or cerebell$ or intracerebral or intracranial or subarachnoid) adj5 (haemorrhage$ or hemorrhage$ or haematoma$ or hematoma$ or bleed$)) or (hemiplegia or paresis) or (hemipleg$ or hemipar$ or paresis or paretic)).kw,ti,ab. (798791)

2 (prognos* or predict*).ti. or ((scor* or risk* or predict* or prognostic*) adj1 (system* or model* or equation* or rule* or tool* or scor*)).ti,kw. or (multivaria* adj1 (scor* or model* or equation* or tool* or rule*)).ti,kw. or "receiver operating characteristic*".ti,kw,ab. or c?statistic.ti,ab,kw. or calibrat*.ti,kw. or validat*.kw,ti. or "roc curve/".kw,ti,ab. (1161591)

3 (stroke or "cerebrovascular diseases" or "age and ageing" or "journal of the american geriatrics society" or neurology or "lancet neurology" or "archives of neurology" or lancet or JAMA or BMJ or "new england journal of medicine").jn. (621638)

4 1 and 2 and 3 (6773)

5 limit 4 to english language (6745)

6 limit 5 to full text (4910)

7 limit 6 to human [Limit not valid in HAPI; records were retained] (4474)

8 limit 7 to humans [Limit not valid in HAPI; records were retained] (4474)

9 limit 8 to yr="2015 -Current" (1335)

# List of journals included in literature search

# BMJ; BMJ Publishing Group limited

- JAMA; American Medical Association
- Lancet; Elsevier Limited
- NEJM; Massachusetts Medical Society
- Cerebrovascular Diseases; Karger International
- Stroke; American Heart Association
- Archives of Neurology; American Medical Association
- Lancet Neurology; Elsevier Limited
- Neurology; American Academy of Neurology
- Age and Ageing; Oxford University Press
- Journal of American Geriatrics Society; John Wiley & Sons
